# The moderating role of gender in the effect of gender perceptions on the sexual satisfaction of married individuals

**DOI:** 10.1186/s40359-025-03736-3

**Published:** 2025-12-03

**Authors:** Selver Bezgin, Elif Solmaz Aşkan, Yener Aktuğ, Murat Örün, Mehmet Tatlı

**Affiliations:** 1 Department of Mental Health and Diseases Nursing, Faculty of Health Sciences, Van Yüzüncü Yıl University, Tuşba, Van, Türkiye; 2 Healthy Life Center, Van Tuşba District Health Directorate, Tuşba, Van, Türkiye; 3 SBU. Van Education and Research Hospital, Van Provincial Health Directorate, İpekyolu, Van, Türkiye; 4 Director of Health Services, Van Provincial Health Directorate, İpekyolu, Van, Türkiye

**Keywords:** Gender perception, Sexual satisfaction, Moderating role, Gender

## Abstract

**Background:**

Turkish society is characterized by a dominant traditional and patriarchal cultural structure. As a result, sexuality is generally considered taboo, and sexual matters are not openly discussed. The aim of this research was to examine the moderating role of gender in the effect of the gender perceptions of married individuals on their sexual satisfaction.

**Methods:**

The current research employed a cross-sectional survey. The Demographic Information Form, Gender Perception Scale, and New Sexual Satisfaction Scale were used as data collection tools. A total of 527 validly completed instruments were used for the data analysis. The moderating effect was assessed using the PROCESS macro created by Hayes [97] for SPSS.

**Results:**

Gender perception and gender had positive and significant effects on sexual satisfaction. It was determined that the moderating effect of gender perception and gender variables on the sexual satisfaction variable was significant.

**Conclusions:**

The study revealed that the impact of gender perception on sexual satisfaction was insignificant for women but significant for men. This finding indicates that gender plays a moderating role in the relationship between gender perception and sexual satisfaction.

## Introduction

The concept of sex refers to the genetic, biological, or physiological attributes of individuals, whereas gender encompasses the social meanings, norms, roles, and behaviors associated with femininity and masculinity [1, 2]. Butler (1999) argues that gender is not an innate or static identity but rather a performance constructed through repeated discourse and behaviors. This perspective highlights that gender is not a natural reality but a socially constructed and continually reproduced structure [3]. Through socialization, individuals learn, internalize, and reproduce gender norms and roles [1]. Gender ideology refers to a system of beliefs about appropriate roles and behaviors for men and women, ranging from traditional to egalitarian perspectives [4], whereas gender perception concerns individuals’ subjective interpretation and evaluation of gender-related attitudes, roles, and norms within their social context [5]. Gender Schema Theory provides a framework for understanding how individuals internalize these norms. It posits that people develop cognitive schemas to make sense of and organize gender roles; these schemas shape how individuals perceive and evaluate themselves and others regarding gender. Societal transmission of gender norms plays a central role in forming these schemas and influencing gender-related attitudes and behaviors [6].

Gender norms are not innate or immutable; they are socially constructed and vary across cultural contexts. These norms play a significant role in shaping the distribution of power, resources, and roles between women and men. Historically, women have occupied a disadvantaged position in many areas [7–9]. This inequality assigns dominant roles such as authority and control to men, while confining women to domestic responsibilities and obedience-based roles [10–14].

Although gender norms have shifted somewhat over time, gender inequality persists [15–19]. According to the United Nations’ Human Development Report on Gender Equality, nearly half of the global population believes that men make better political leaders, over 40% believe men are better business managers, and 28% think a man has the right to beat his wife [20]. The World Economic Forum’s Global Gender Gap (GGG) Report indicates that, while Western Europe has seen the greatest reduction in inequality (77.6%), the Middle East and North Africa (including Türkiye) have seen the least (60.9%). Türkiye’s ranking in the global gender index dropped from 115th in 2006 to 133rd in 2021 [21].

Gender inequality adversely affects couple dynamics within marriage and can result in psychological problems for individuals [7–9, 22–32]. Research indicates that while the physical and psychological impacts of inequality disproportionately affect women, men also suffer significant psychological consequences [7–9, 24–34]. This inequality extends into the realms of sexuality and sexual satisfaction, adversely affecting sexual health [35]. Meta-analyses and systematic reviews report that sexual dysfunction is more prevalent among women, especially in societies with pronounced gender inequality [36, 37]. In male-dominated cultures, the sexual desire gap between women and men becomes more evident, suggesting that such disparities are often shaped by cultural norms and power imbalances rather than biological factors [38, 39].

Sexual satisfaction is an emotional response that reflects a person’s subjective evaluation of their sexual life and describes the pleasure and fulfillment derived from sexual relationships [40, 41]. It fosters intimacy, trust, and closeness between partners, and contributes to overall marital and life satisfaction [42–47]. Additionally, it impacts individual and family physical and mental health and is associated with broader social and economic development [39].

In developing countries, cultural norms frequently frame sexuality as a private or sensitive issue, resulting in inadequate investigation of sexual health and related challenges [48, 49]. In Türkiye, social and cultural structures sustain the taboos surrounding sexuality, and open discussion remains rare [50, 51]. Though comprehensive data are lacking, sexual problems are believed to be common among both women and men [52]. Studies in Türkiye indicate that women report more sexual problems than men, men report higher levels of sexual satisfaction, and adherence to traditional gender roles correlates with lower sexual satisfaction [50, 53–56]. A growing body of research demonstrates that sexual roles attributed within the context of gender inequality and the pressures those roles engender negatively influence sexual desire and satisfaction [35, 57–71]. However, the literature remains inconsistent on gender’s role in this dynamic. Some studies identify significant differences between men and women [70–73, 76, 77], while others emphasize similarities [75, 78–80]. Meta analyses by Petersen and Hyde (2010, 2011) conclude that male and female sexuality are largely similar, with observed differences primarily shaped by gender roles and stereotypes [75, 79]. Literature also includes investigations of gender as a potential moderator in the relationship between sexual satisfaction and quality of life, for example, Stephenson et al. (2021) and Stelmar et al. (2024) examine this interplay from a gender perspective [77, 81], alongside research evaluating gender perception within gendered contexts [82–84]. In conclusion, gender roles and attitudes toward sexuality significantly shape individuals’ sexual lives and psychological well being. However, the extent to which gender perceptions influence sexual satisfaction differently for men and women remains unclear.

**Fig. 1:**
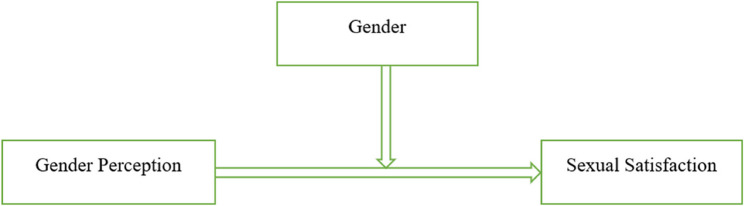
Conceptual model

Sexual Script Theory provides a crucial theoretical framework for explaining the role of gender in the relationship between gender perceptions and sexual satisfaction. This theory posits that sexual behaviors are shaped not only by biological drives but also profoundly influenced by culturally and socially constructed scripts. These scripts assign active roles to men and passive roles to women based on traditional gender roles, thereby differentiating expectations, behavioral patterns, and evaluative criteria related to sexual experiences between women and men [85]. Additionally, Gender Schema Theory offers a conceptual perspective for understanding how these gendered scripts are internalized by individuals [6]. Together, these theories not only elucidate how cultural and social norms shape sexual behaviors and experiences but also suggest that internalized gender roles can affect how sexual satisfaction is perceived and evaluated (Fig. 1).

Given that men and women are typically socialized into distinct gender roles, leading to the development of different cognitive schemas and sexual scripts, it is plausible that gender plays a moderating role in the relationship between gender perceptions and sexual satisfaction. Based on this theoretical foundation, this study aims to examine whether gender plays a moderating role in the relationship between married individuals’ gender perceptions and their levels of sexual satisfaction. For this purpose, the following hypothesis was tested:

- H1: Gender has a moderating role in the effect of gender perceptions on the sexual satisfaction of married individuals.

## Methods

A cross-sectional survey, a prevalent quantitative research approach, was employed in the current research. Such a survey collects data from a sample chosen from a defined demographic. Furthermore, the data are collected at a certain point in time, even if the gathering procedure may extend from a single day to several weeks or more [86].

### Participants

The population of this research consisted of married individuals living in Türkiye. According to the Turkish Statistical Institute (TÜİK), there were 40,220,950 married individuals in Türkiye in 2023 (foreigners were not included) [87]. The G-power program was used to estimate a minimum sample size of 395 participants (*F* test, 5% multiple regressions, fixed model, *R*^*2*^ increase, ⍺ error = 5%, power = 80%). Nevertheless, to enhance the robustness of the findings, data from 527 married individuals—recruited through convenience sampling—were collected and analyzed.

The inclusion criteria for participants were defined as residing in Türkiye, being married, possessing at least basic literacy skills, and, for female participants, being between 18 and 55 years of age (considering the reproductive period). Exclusion criteria applicable to all participants included being a foreign national, having any chronic illness, or having a diagnosis of depression or another psychiatric disorder. Additionally, exclusion criteria specific to women included pregnancy, the postpartum period, and being in the menopausal stage. The demographic information of the participants is presented in Table 1.

**Table 1:** Participants’ demographic information (*N* = 527)

**Variable**		**n**			**%**
Gender	Female	342			64.9
	Male	185			35.1
		Female (n)	%	Male (n)	%
Age (Years)	20-25	43	12.6	10	5.4
	26-30	65	19.0	50	27.0
	31-35	96	28.1	28	15.1
	36-40	54	15.8	34	18.4
	41 ≥	84	24.6	63	34.1
Employment situation	Does not work	193	56.4	4	2.2
	Public servant	112	32.7	109	58.9
	Private sector	37	10.8	72	38.9
Education level	Elementary/Middle School	74	21.6	11	5.9
	High school	64	18.7	52	28.1
	University	204	59.6	122	65.9
The settlement where they grew up	Village	48	14.0	33	17.8
	District	108	31.6	55	29.7
	Province	186	54.4	97	52.4
Financial income	Insufficient	156	45.6	102	55.1
	Sufficient	186	54.4	83	44.9
Type of marriage	Arranged marriage	127	37.1	32	17.3
	Marriage through dating	215	62.9	153	82.7
Duration of marriage (Years)	0-5	115	33.6	75	40.5
	6-12	116	33.9	30	16.2
	13-19	49	14.3	34	18.4
	20 ≥	62	18.1	46	24.9
Number of children	1	74	21.6	17	9.2
	2	178	52.0	138	74.6
	3 ≥	90	26.3	30	16.2

### Data collection process

Following ethical approval, married adults identified through convenience sampling based on the research objectives were reached via social media platforms such as WhatsApp and Facebook. Participants were informed about the study. Informed consent was obtained via a form presented at the beginning of the online data collection instrument (Google Forms). In total, 541 participants voluntarily completed the subsequent data collection tools between June and September 2024. However, the answers of 14 participants who were not eligible were not considered. Therefore, 527 validly completed instruments were used for the data analysis.

### Data collection tools

#### Demographic information form

This research employed a demographic questionnaire to collect relevant personal information from the participating married individuals. The instrument included items pertaining to gender, age, employment status, educational attainment, place of upbringing, financial income, type of marriage, marital duration, and number of children. As related to type of marriage; arranged marriage refers to a form of union in which two individuals marry without having met each other directly, typically facilitated by a third party such as elder family members. This type of marriage is commonly practiced in traditional Turkish society. In contrast, marriage through dating denotes a union where couples marry based on mutual consent and personal choice after getting to know each other. The variable of marital duration was categorized into 0–5, 6–12, 13–19, and 20 years or more, taking into account the family life cycle theory [88, 89] and empirical studies demonstrating changes in sexual and marital satisfaction over the course of marriage [90–94].

#### Gender perception scale

This scale was created by Altınova and Duyan (2013) to assess individuals’ gender perceptions. The Gender Perception Scale has 25 items on a 5-point Likert scale (1 = *strongly disagree*, 5 = *strongly agree*). The scale comprises positive statements, including “Marriage does not inhibit women from working” and “A working woman can adequately spend time with her children” (10 items), alongside negatively scored statements, such as “A woman should refrain from working if her husband disapproves” and “A woman without a husband resembles a house without an owner” (15 items). The minimum obtainable score on the scale is 25, and the maximum is 125. Elevated scores on the measure signify an egalitarian perception of gender roles. The minimum score of 25 was subtracted from the maximum score of 125, yielding a result of 100. The score of 100 was segmented into three categories: individuals scoring between 25 and 58 were classified as possessing low gender perception, those scoring between 59 and 92 were classified as having medium gender perception, and individuals scoring between 93 and 125 were classified as exhibiting high gender perception. The authors of this study developed this three-level classification. Participants with high scores exhibit a positive or egalitarian gender perception, those with medium levels demonstrate a moderate gender perception, and those with low scores reflect a negative or unequal gender perception. The descriptive data concerning current participants’ gender perceptions are as follows: Low *n* = 48, 9.1%; Medium *n* = 158, 30%; High *n* = 321, 60.9%. An exploratory factor analysis performed to assess the scale’s validity revealed that it comprised a singular dimension. The scale had a Cronbach’s alpha reliability coefficient of 0.87 [5]. The Cronbach’s alpha reliability coefficient of the scale in this research was 0.75.

#### New sexual satisfaction scale (NSSS)

The Turkish validity and reliability of the scale, developed by Stulhofer et al. (2010), were assessed by Tuğut (2016). The instrument designed to assess sexual pleasure in clinical and field studies is a 5-point Likert-type evaluation tool. The minimum score on the scale is 20, while the maximum score is 100. The scale comprises two subdimensions: self-centered and partner- or sexual activity-centered. In the self-centered subdimension of the scale, items such as “My emotional openness during sexual intercourse” are included, while in the partner- or sexual activity-centered subdimension, items such as “My partner’s sexual creativity” are present. The self-centered subdimension assesses sexual satisfaction derived from individual experiences and feelings. The partner- or sexual activity-centered subdimension assesses an individual’s sexual pleasure derived from their partner’s sexual behaviors and responses, along with the variety or frequency of sexual activities. The scale’s score is determined by summing the components. Elevated scores on the measure signify favorable sexual pleasure. The Cronbach’s alpha coefficient for the whole scale was 0.94 [95, 96]. This research computed the Cronbach’s alpha coefficient as 0.95 for the overall scale. In this research, the aggregate score was used.

### Data analysis

The descriptive features of the research participants were determined using frequency and percentage values. Prior to the statistical analysis, the assumptions of the moderator analyses were examined. Consequently, the data must conform to univariate and multivariate normality. Furthermore, the dataset must be devoid of multicollinearity issues. A correlation value of 0.30 or below is considered low correlation, between 0.30 and 0.70 is regarded as moderate correlation, and 0.70 or more is classified as high correlation [97]. The dataset was analyzed with the SPSS-27 software package. Sociodemographic variables (e.g., age, education, and marital duration) were not statistically controlled for in the analysis. To test the moderating role of the gender variable in the effect of married individuals’ gender perceptions on their sexual satisfaction, a regression analysis was conducted using Process Macro developed by Hayes (2018) for SPSS [98]. This research employed Model 1 to ascertain the moderating effect.

## Results

### Descriptive statistics and interrelationships among variables

Table 2 presents the mean, standard deviation, minimum and maximum scores, and Pearson’s product-moment correlation coefficients. There was a moderate positive correlation between gender perception and sex satisfaction (*r* = 0.38, *p* < 0.01).

**Table 2:** Descriptive statistics and correlation analysis for the variables (*N* = 527)

	Variable	Descriptive statistics						Correlation coefficients	
		Min.	Max.	Mean	Std. Dev.	Skewness	Kurtosis	1.	2.
Female	1. GP	30.00	125.00	95.92	20.06	− .802	.098	-	.068
	2. SS	20.00	100.00	76.15	17.35	− .852	.554		-
Male	1. GP	33.00	122.00	90.75	25.77	− .777	− .699	-	.721**
	2. SS	41.00	100.00	81.57	19.10	− .649	−1.068		-
Total	1. GP	30.00	125.00	94.11	22.35	− .873	− .073	-	.328**
	2. SS	20.00	100.00	78.05	18.16	− .703	− .134		-

*GP* *Gender perception, SS* *Sex satisfaction*

^**^p<0.01

### The moderating role of gender

The regression analysis results are given in Table 3. According to the results in Table 3, the predictor variable included in the regression analysis explained approximately 22% (*R*^*2*^ = 0.224) of the change in the sexual satisfaction variable. It was determined that gender perception (*b* = 0.225, *p* < 0.01) and gender (*b* = 7.32, *p* < 0.01) had positive and significant effects on sexual satisfaction. It was determined that the interactive effect (moderating effect) of gender perception and gender variables on the sexual satisfaction variable was significant (*b* = 0.475, *p* < 0.01).

**Table 3:** Regression analysis results showing the moderating effect (*N* = 527)

Variables	Standardized Coefficients (β)	Unstandardized Coefficients (B)	Standard Error	t
Constant	.030 [0.04, 0.10]	78.613*** [77, 80]	.702	111.91
Gender Perception (X)	.277 [0.19, 0.35]	.225*** [0.16, 0.29]	.032	6.96
Gender (W)	.403 [0.24, 0.56]	7.323*** [4.42, 10]	1.473	4.96
X.W	.585 [0.43, 0.73]	.475*** [0.35, 0.60]	.063	7.54

*R* = 0.473, *R*^2^ = 0.224, ^***^p < 0.001, ^**^p < 0.01, ^*^p < 0.05

**Fig. 2:**
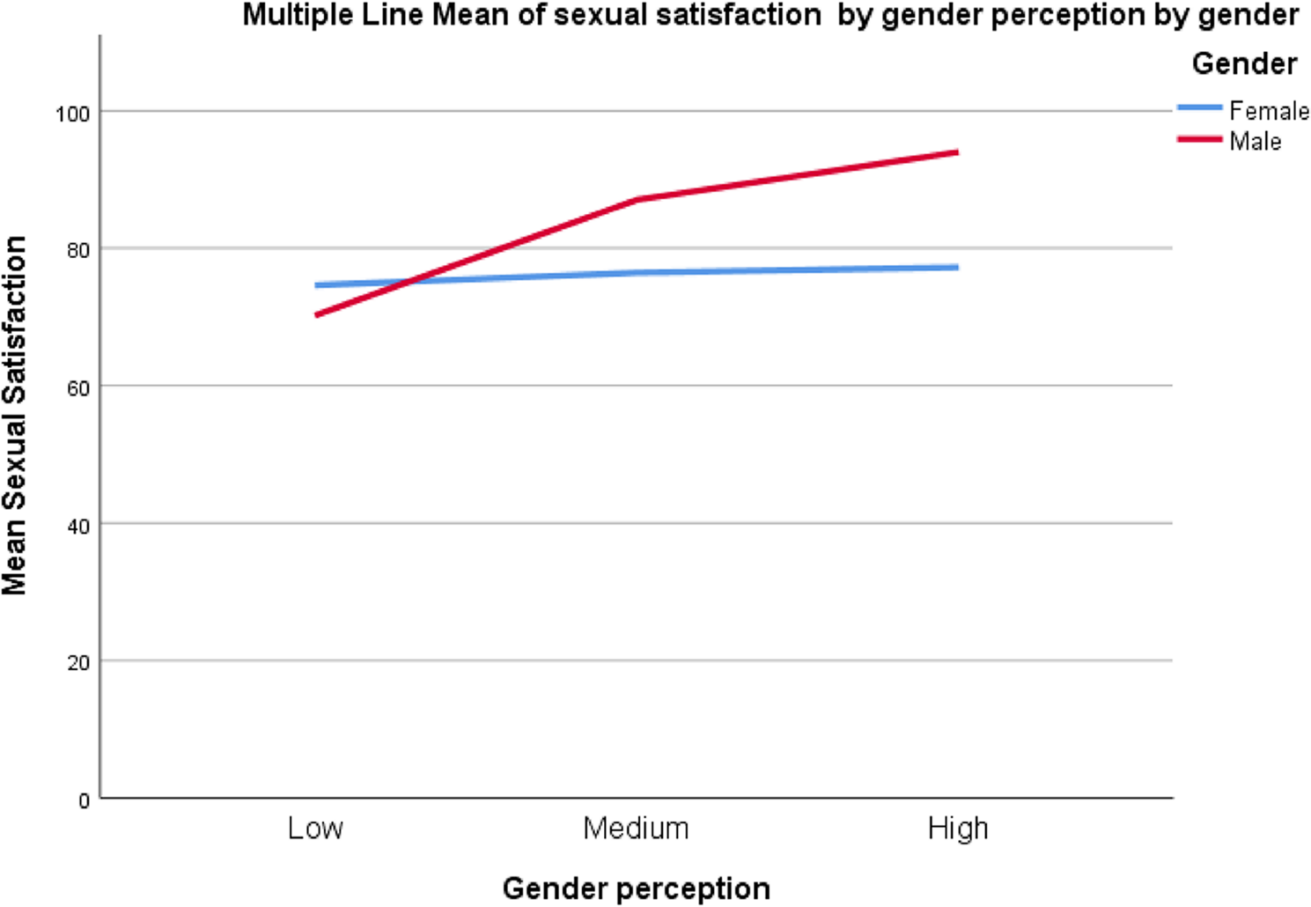
Multiple line mean of sexual satisfaction by gender perception by gender

Slope analysis was used to understand the effects of the moderator variable, as shown graphically in (Fig. 2). Gender perception was divided into three categories: low, medium, and high. It was determined that the effect of gender perception on sexual satisfaction was not significant in women (*b* = 0.058, *p* = 0.176). In men, the effect of gender perception on sexual satisfaction was found to be significant (*b* = 0.534, *p* < 0.001). These findings confirmed *H1*.

The relationships between participants’ sociodemographic variables and sexual satisfaction were examined using ETA correlation analysis. The results indicated a weak correlation between age (η =.372, p <.05), duration of marriage (η =.360, p <.05), and education level (η =.235, p <.05) and sexual satisfaction. In contrast, no significant correlation was found between sexual satisfaction and the variables ‘‘the settlement where participants grew up’’ (η =.146, p >.05) ‘‘type of marriage’’ (η =.151, p >.05), and ‘‘number of children” (η =.193, p >.05) (Table 4).

**Table 4:** Correlation analysis between sociodemographic variables and sexual satisfaction (N = 527)

Variables	Sexual satisfaction
Age	.372*
Marital duration	.360*
Education level	.235*
The settlement where they grew up	.146
Type of marriage	.151
Number of children	.193

^*^weak association

## Discussion

This study explores whether gender moderates the relationship between gender perceptions and sexual satisfaction among married individuals. The findings indicate that gender perception significantly influences sexual satisfaction in males, but not in females, suggesting that gender serves as a moderating variable in this relationship. Consistent with prior research, gender has been shown to affect both sexual satisfaction and perceptions of gender roles [77, 81–84]. Several studies have demonstrated that men and women differ in their sexual desires and expectations of fulfillment. Specifically, women’s sexual desire and satisfaction are more closely associated with love and emotional intimacy, whereas men’s are generally more mechanical and performance-oriented [73, 77, 99, 100].

In the present study, the significant influence of gender perception on sexual satisfaction among men—but not women—may be explained by the persistence of traditional gender norms. Men, who are culturally encouraged to engage with and value sexuality more openly than women, may be more acutely aware of the negative impact of gender norms on their sexual satisfaction [67, 101]. Previous research has suggested that masculinity-oriented cultural messages strongly shape men’s sexual function and experiences of pleasure [61, 67–69, 71]. In contrast, some women may internalize traditional sexual norms, which may account for the lack of a significant relationship between gender perception and sexual satisfaction. In patriarchal societies such as Turkey, women are often taught to ‘‘suppress sexual desire until marriage and to satisfy their husbands sexually thereafter’’ [62, 102, 103]. Numerous studies have shown that many women adopt these roles unconsciously [67, 101, 103–105]. For example, a study of 102 Turkish women found that, due to cultural expectations, many women in Turkey do not perceive sexual intercourse as a right and view sexual inactivity after a certain age as natural [104]. An international study also revealed that, in men, the frequency of sexual intercourse is directly related to their own episodes of arousal, whereas in women, it is more strongly linked to their partner’s arousal [67].

The current findings show that perceptions of gender roles do not significantly affect sexual satisfaction in women, potentially due to their restricted sexual experiences and limited sexual awareness [67, 101, 103, 105]. Multiple Turkish studies have demonstrated that, even in contexts with relatively egalitarian gender norms, women report lower levels of sexual pleasure than men [50, 54]. This may be attributed to a lack of comprehensive understanding of sexuality among women [106, 107]. Indeed, research indicates that even highly educated individuals in Turkey often possess limited knowledge about sexuality, and that such knowledge is positively associated with higher sexual satisfaction [23, 106, 107]. The absence of comprehensive sex education within Turkey’s formal education system, coupled with the inadequacy of existing premarital sexual health education, exacerbates the overall lack of sexual knowledge [108].

The minimum score attained by men in this study on the gender perception scale was 33, whilst the maximum was 122. Men’s low or high gender perception scores can typically be attributed to gender roles, sexual education, personal growth, and psychological variables. This pertains directly to an individual’s consciousness of their own gender and sexuality.

Sexual satisfaction is a multifaceted concept that transcends gender perception and is influenced by numerous aspects, including age, marital duration, educational attainment, personality features, and personal beliefs, especially regarding sexual knowledge and awareness [23, 106, 109, 110]. The present study also found weak positive relationships among age, duration of marriage, education level, and sexual satisfaction. As individuals age and their marriages evolve, they get greater familiarity with their partners. Individuals can exhibit greater transparency and candor regarding sexual preferences, requirements, and communication. This may result in enhanced harmony and fulfillment in their sexual lives over time. Understanding that sexuality serves as a conduit for emotional connection rather than solely a physical interaction, and engaging more deliberately in this context, can enhance satisfaction. An elevated level of education typically signifies that individuals exhibit superior communication abilities and a greater propensity for open-mindedness. Educated persons may possess more awareness of sexual health, partner communication, and emotional intelligence. This insight can facilitate more effective discussions regarding their sexual desires with partners and identify solutions to future issues.

Regarding the nonsignificant relationship between gender perception and sexual satisfaction in women found in this study, this result may also stem from limitations in the measurement tools and methodologies used. For instance, some female participants may have underestimated their levels of sexual satisfaction due to societal norms and self-report biases [74], thereby complicating the statistical evaluation of the associations between variables. Furthermore, the limited variability in gender perception scores—indicating homogeneity in participants’ gender role attitudes—may have attenuated the observed relationship with sexual satisfaction.

In conclusion, the key findings of this study indicate that the influence of gender perception on sexual satisfaction varies with gender. Although a significant relationship was found between gender perception and sexual satisfaction in men, no such significant relationship was found among women. This result suggests that gender plays a moderating role in the relationship between gender perception and sexual satisfaction. The study results contribute substantially to deepening our understanding of the influence of gender on the relationships between gender perception and sexual satisfaction—especially in patriarchal settings. Consequently, broadening sexuality education with a comprehensive framework that takes cultural context into account is needed not only to fill the current knowledge gaps but also to improve the individual’s ability to identify and articulate their sexual rights. Revamping school-based sexual health education programs and premarital counseling services to incorporate information on gender disparities can be a beneficial intervention, especially in enhancing women’s sexual awareness and mitigating gender-based structural inequities.

Promoting egalitarian gender perceptions plays a central role in improving the scope and effectiveness of sexual health interventions. For women, it can support the development of self-confidence in recognizing and asserting their sexual rights. For men, it may help alleviate performance-related pressures arising from traditional gender norms and foster a more balanced, emotionally connected sexual experience. As a result, it becomes possible to design inclusive and holistic interventions that are responsive to the needs of both women and men. Furthermore, enacting legal and institutional policies that promote gender equality may facilitate enduring structural changes that enhance individuals’ sexual health and enjoyment.

### Limitations of the study

This study has several limitations that must be taken into consideration when interpreting its findings. First, the sample comprised only married individuals residing in Turkey. This limits the generalizability of the findings to individuals from different cultural backgrounds. The sexual satisfaction experienced by married individuals is shaped by the complex interaction of multiple factors, such as age (the physical and psychological changes that accompany aging), education (sexual knowledge and sexual awareness), and duration of marriage (relationship dynamics and routinization). Each of these variables—alone or in combination with others—can positively or negatively affect sexual satisfaction. In this study, sociodemographic variables, such as age, level of education, and duration of marriage, were not statistically controlled for in the analysis. Additionally, applying an age limit only to women, based on reproductive considerations, may introduce methodological bias in gender comparisons. This may potentially be considered a limitation that affects the validity of the findings. The study examined the employment status variable but omitted variables such as employment type or sector of employment. The study concentrated on gender; hence these characteristics were not considered. This may be considered as a limitation of the study. Moreover, the use of self-report measures for data collection increases the risk of social desirability bias, especially in cultural contexts where sexuality is considered a sensitive and stigmatized topic. Also, regarding the nonsignificant the effect of gender perception on sexual satisfaction in women found in this study, this result may also stem from limitations in the measurement tools and methodologies used. Future research that addresses these limitations may yield more robust and generalizable findings. Also, future research should include not only married persons or solely the Turkish context but also include a wider array of sample groups and participants from different countries.

## Data Availability

Due to confidentiality/ethical restrictions, the data are not being shared.
